# A new ankylosaurid skeleton from the Upper Cretaceous Baruungoyot Formation of Mongolia: its implications for ankylosaurid postcranial evolution

**DOI:** 10.1038/s41598-021-83568-4

**Published:** 2021-03-18

**Authors:** Jin-Young Park, Yuong-Nam Lee, Philip J. Currie, Michael J. Ryan, Phil Bell, Robin Sissons, Eva B. Koppelhus, Rinchen Barsbold, Sungjin Lee, Su-Hwan Kim

**Affiliations:** 1 School of Earth and Environmental Sciences, Seoul National University, Seoul, 08826 South Korea; 2 Department of Biological Sciences, CW 405 Biological Sciences Building, University of Alberta, Edmonton, AB T6G 2E9 Canada; 3 Department of Earth Sciences, Carleton University, 2125 Herzberg Building, 1125 Colonel By Drive, Ottawa, ON K1S 5B6 Canada; 4 Department of Palaeobiology, Canadian Museum of Nature, Station ‘D’, P.O. Box 3443, Ottawa, ON K1P 6P4 Canada; 5 Palaeoscience Research Centre, University of New England, Armidale, NSW 2351 Australia; 6 Institute of Paleontology, Mongolian Academy of Sciences, Box-46/650, Ulaanbaatar, 15160 Mongolia

**Keywords:** Evolution, Palaeontology

## Abstract

A new articulated postcranial specimen of an indeterminate ankylosaurid dinosaur from the Upper Cretaceous (middle-upper Campanian) Baruungoyot Formation from Hermiin Tsav, southern Gobi Desert, Mongolia includes twelve dorsal vertebrae, ribs, pectoral girdles, forelimbs, pelvic girdles, hind limbs, and free osteoderms. The new specimen shows that Asian ankylosaurids evolved rigid bodies with a decreased number of pedal phalanges. It also implies that there were at least two forms of flank armor within Ankylosauridae, one with spine-like osteoderms and the other with keeled rhomboidal osteoderms. Unique anatomical features related to digging are present in Ankylosauridae, such as dorsoventrally flattened and fusiform body shapes, extensively fused series of vertebrae, anteroposteriorly broadened dorsal ribs, a robust humerus with a well-developed deltopectoral crest, a short robust ulna with a well-developed olecranon process, a trowel-like manus, and decreased numbers of pedal phalanges. Although not fossorial, ankylosaurids were likely able to dig the substrate, taking advantage of it for self-defence and survival.

## Introduction

Ankylosaurs were herbivorous quadrupedal dinosaurs characterized by heavily ornamented skulls and transverse rows of osteoderms that covered the dorsolateral surfaces of their bodies^1^. Abundant remains of ankylosaurs have been found in Mongolia, and a total of nine taxa are currently known (Table 1). However, most of the taxa are based on skulls, whereas articulated postcranial specimens are scarce, with only three specimens currently described in the scientific literature^2–4^. There are nearly complete postcranial skeletons of *Pinacosaurus grangeri* (PIN 614) and an indeterminate ankylosaurid (MPC 100/1305) from the Djadokhta Formation (Campanian), and the holotype of *Saichania* (MPC 100/151), which comprises a skull with the anterior portion of the postcranial skeleton.

**Table 1:** Ankylosaur taxa of Mongolia.

Taxa	Occurrence
Ankylosauridae Brown^6^	
Shamosaurinae Tumanova^56^	
*Shamosaurus* Tumanova^56^	
*Sh. scutatus* Tumanova^56^	Zuunbayan Formation (Aptian–Albian)
Ankylosaurinae Nopcsa^57^	
*Minotaurasaurus* Miles and Miles^58^	
*M. ramachandrani* Miles and Miles^58^	Djadokhta Formation (Campanian)
*Pinacosaurus* Gilmore^59^	
*P. grangeri* Gilmore^59^	Djadokhta Formation (Campanian)
*Saichania* Maryañska^3^	
*Sa. chulsanensis* Maryañska^3^	Baruungoyot Formation (middle-upper Campanian)
*Talarurus* Maleev^60^	
*Tal. plicatospineus* Maleev^60^	Bayanshiree Formation (Cenomanian-Santonian)
*Tarchia* Maryañska^3^	
*Tar. kielanae* Maryañska^3^	Baruungoyot Formation (middle-upper Campanian)
*Tar. teresae* Penkalski and Tumanova^15^	Nemegt Formation (upper Campanian-lower Maastrichtian)
*Tsagantegia* Tumanova^61^	
*Ts. longicranialis* Tumanova^61^	Bayanshiree Formation (Cenomanian-Santonian)
*Zaraapelta* Arbour et al.^12^	
*Z. nomadis* Arbour et al.^12^	Baruungoyot Formation (middle-upper Campanian)

**Figure 1:**
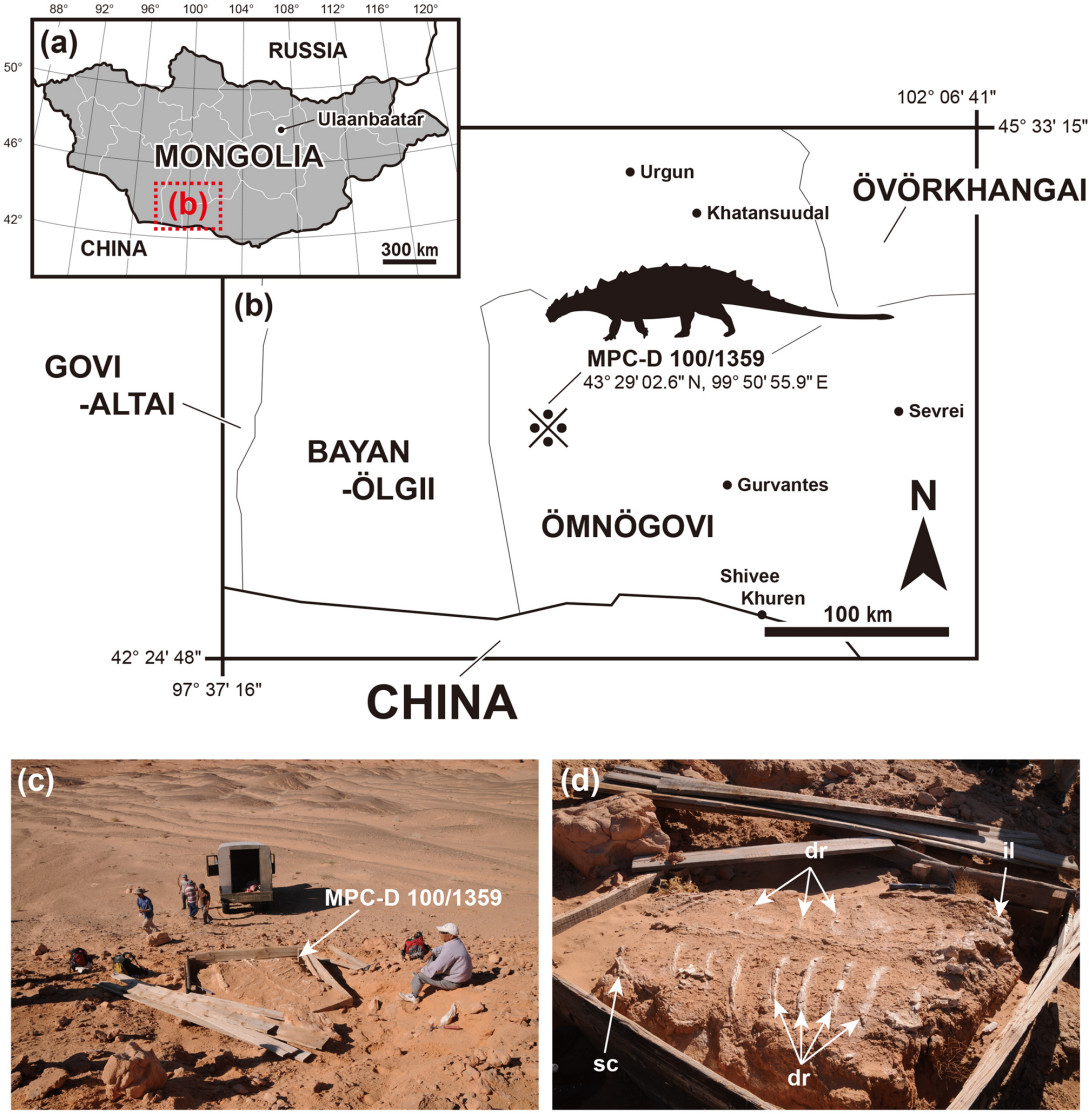
Map showing where the new ankylosaurid postcranial specimen (MPC-D 100/1359) was discovered. (**a**) Map of Mongolia. (**b**) Enlarged map of the rectangle with the dotted line in (**a**). The fossil locality is marked by a symbol (※). (**c**) Photograph of the excavation site. (**d**) Close up photograph of the exposed dorsal surface of the specimen. Adobe Illustrator CC (version 24.0.1, https://www.adobe.com/kr/products/illustrator.html) was employed to produce (**a**, **b**).

In 2008, an articulated postcranial material (MPC-D 100/1359) was collected from the Upper Cretaceous (middle-upper Campanian) Baruungoyot Formation at Hermiin Tsav by members of Korea-Mongolia International Dinosaur Expedition (Fig. 1). It was first discovered and uncovered by the Joint Soviet-Mongolian Paleontological Expedition team in 1972 or 1973 (Ligden Barsbold, personal communication May 20, 2020). They prepared it for excavation as a monolith, but ran out of materials and time, and abandoned it with the intention of excavating it at a later time. As far as we know, it was next seen by a Dinosaurs of the Gobi (Nomadic) Expedition in 1999, but it had no doubt been seen by many other expeditions in the meantime. At that time, the sides of the specimen were still surrounded by a wooden crate, but the top was covered by loose boards. All of the intervening spaces had been filled in by loose, wind-blown sand. It is uncertain whether there was a skull and/or a tail club associated with the postcranial skeleton, although it’s unlikely the skull was present because the cervical half-rings are missing. The specimen includes twelve dorsal vertebrae, ribs, pectoral girdles, forelimbs, pelvic girdles, hindlimbs, and free osteoderms, which present the most complete postcranial skeleton of an ankylosaurid from the Baruungoyot Formation. Therefore, this articulated postcranial skeleton with in situ dermal scutes provides valuable insight into the postcranial evolution of ankylosaurids. It also provides insight based on anatomical features into the possibility of ankylosaurid digging behavior.

### Institutional abbreviations

**MPC**, Mongolian Paleontological Center, Mongolian Academy of Sciences, Ulaanbaatar, Mongolia; **PIN**, Paleontological Institute, Russian Academy of Sciences, Moscow, Russia; **ZPAL**, Zaklad Paleobiologii (Institute of Paleobiology)–Polish Academy of Sciences, Warsaw, Poland.

## Results

### Systematic paleontology

Dinosauria Owen^5^.

Ankylosauridae Brown^6^.

Ankylosauridae, gen. et sp. indet.

### Material

**Figure 2:**
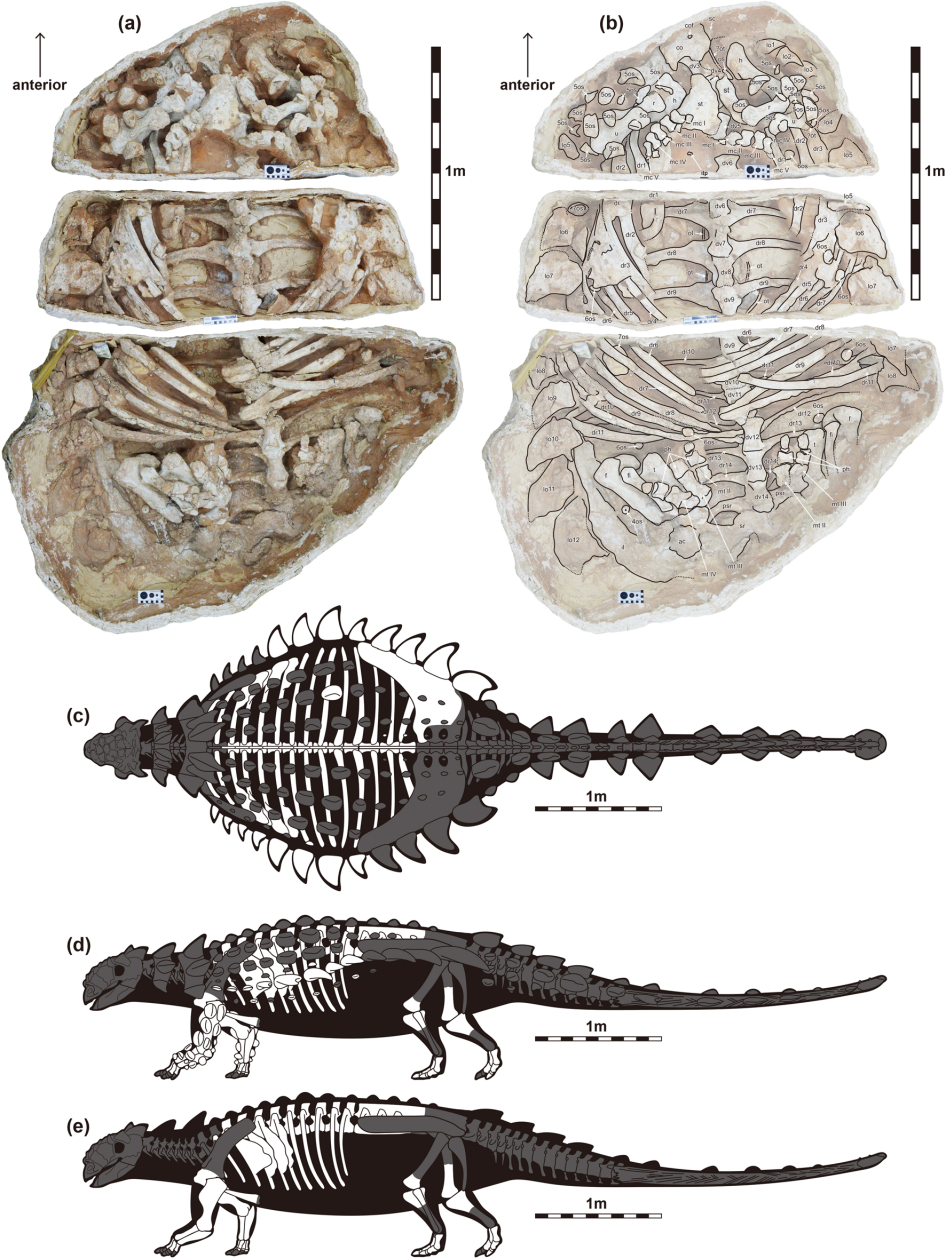
The new ankylosaurid postcranial specimen (MPC-D 100/1359). (**a**) Photograph and (**b**) line drawing of the specimen in ventral view. (**c**–**e**) Skeletal diagram of the specimen in dorsal view (**c**), left lateral views with dermal armor (**d**) and without dermal armor (**e**). Abbreviations: 4os, Type 4 osteoderm; 5os, Type 5 osteoderm; 6os, Type 6 osteoderm; 7os, Type 7 osteoderm; ac, acetabulum; co, coracoid; cof, coracoid foramen; dr, dorsal rib; dv, dorsal vertebra; f, femur; fi, fibula; h, humerus; il, ilium; itp, isolated theropod phalanx; lo, lateral osteoderm; mc, metacarpal; mt, metatarsal; ot, ossified tendon; psr, parasacral rib; ph, phalanx; r, radius; sc, scapula; sr, sacral rib; st, sternal plate; sv, sacral vertebra; t, tibia; u, ulna. Adobe Illustrator CC (version 24.0.1, https://www.adobe.com/kr/products/illustrator.html) was employed to produce (**c**–**e**).

**Figure 3:**
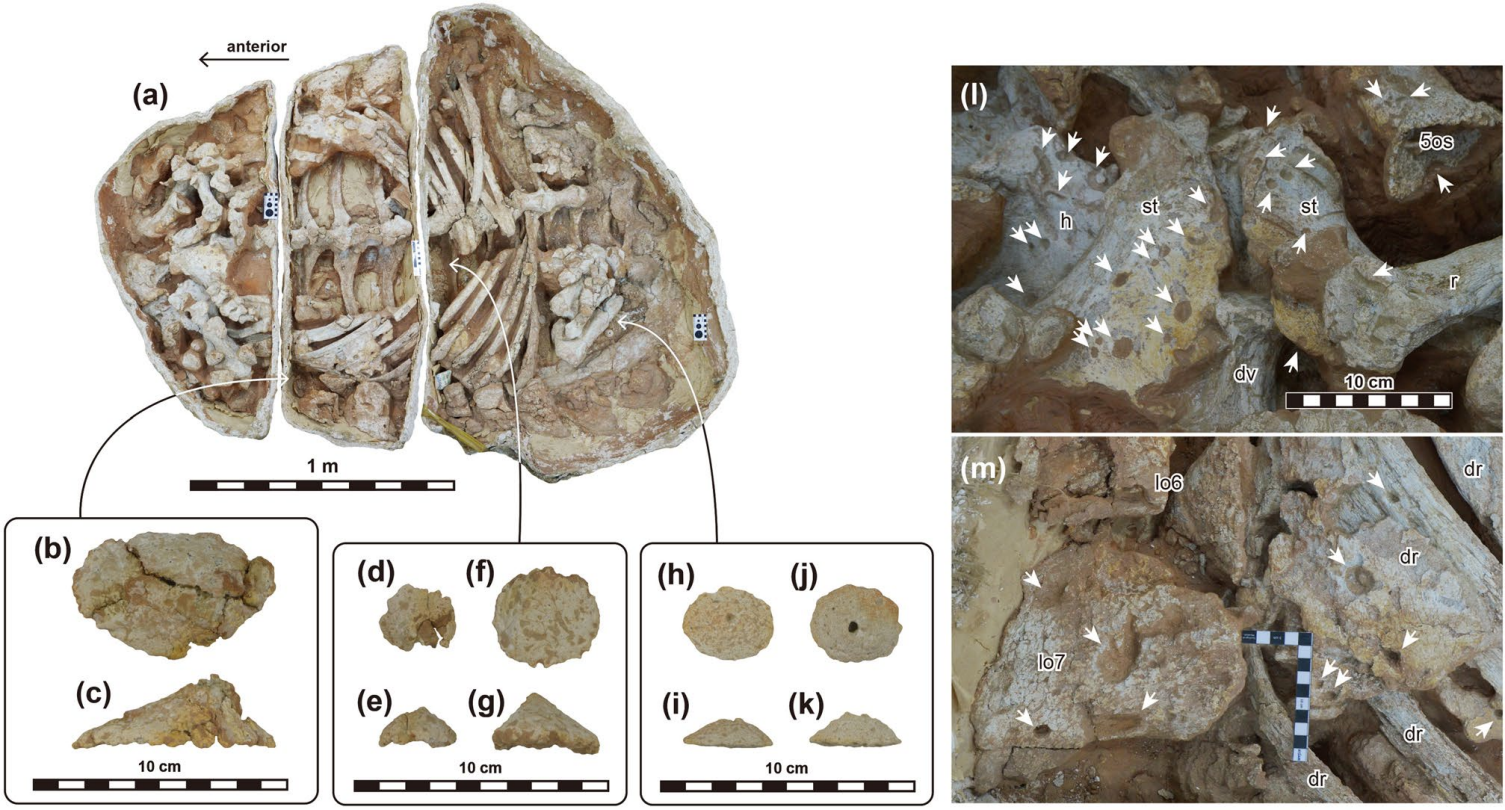
Selected osteoderms (**a**–**k**) and feeding traces of dermestid beetles (**l** and **m**). (**a**) MPC-D 100/1359 in ventral view. Arrows indicate the areas where the selected osteoderms were collected. (**b** and **c**) Type 7 osteoderm in lateral (**b**) and dorsal (**c**) views. (**d**–**k**) Type 4 osteoderms in lateral (**d**, **f**, **h**, **j**) and dorsal (**e**, **f**, **i**, **k**) views. (**l**) Close up photo of sternal plates. (**m**) Close up photo of the right lateral edge of the middle portion of the trunk. White arrows mark feeding traces. Abbreviations: 5os, Type 5 osteoderm; dr, dorsal rib; dv, dorsal vertebra; h, humerus; lo, lateral osteoderm; r, radius.

MPC-D 100/1359 (Figs. 1, 2, 3), a nearly complete postcranial skeleton that lacks the tail but includes twelve dorsal vertebrae, ribs, pectoral girdles, forelimbs, pelvic girdles, hindlimbs, and free osteoderms.

### Locality and horizon

Aeolian red sandstone deposit of the Upper Cretaceous (middle-upper Campanian) Baruungoyot Formation^7^, Hermiin Tsav, southern Gobi Desert, Mongolia.

### Remarks

The presence of a synsacrum, the horizontal rotation of the ilium, closed acetabulum, absence of the pubis, and dermal armor on the lateral surface of the body clearly shows that MPC-D 100/1359 belongs to Ankylosauria^8,9^. Furthermore, MPC-D 100/1359 contains ankylosaurid features such as a broad humerus, unguals that are wider than long, and a tridactyl pes^8,10,11^. A total of three ankylosaurid taxa – *Saichania chulsanensis*, *Tarchia kielanae*, and *Zaraapelta nomadis* – are known from the Baruungoyot Formation^3,12^. *Saichania* is similar to MPC-D 100/1359 in having broad but irregular, plate-like, bony intercostal processes on the dorsal ribs, and a robust ulna. However, *Saichania* differs from MPC-D 100/1359 by having fused dorsal ribs from the sixth dorsal vertebra, bony processes starting from the fifth rib, medially fused sternal plates, more robust humeri, a spur-like lateral process on the proximal end of the humerus, a pointed olecranon on the ulna, lateral and medial femoral condyles that have the same heights, and three rows of spine-like flank osteoderms (two rows of dorsoposteriorly bent osteoderms, and ventral to them, one row of dorsoanteriorly bent osteoderms)^3,13,14^. No postcranial materials are known from *Tarchia kielanae* and *Zaraapelta*, so comparisons to MPC-D 100/1359 are currently not possible^3,12,15^. For these reasons, MPC-D 100/1359 must be considered as an indeterminate ankylosaurid.

### Description

The body lies horizontally in a “resting posture”, with both forelimbs and hindlimbs folded and tucked underneath the torso (Fig. 2). The preserved trunk is 203 cm in length and 129 mm in greatest width (external osteoderms are excluded, see Supplementary Information for detailed measurements). Feeding traces of dermestid beetles can be observed on the dorsal ribs, sternal plates, forelimbs, and dermal armor (Fig. 3l,m). These circular borings are 5.5 to 36.25 mm in diameter. Five small, isolated theropod phalanges were also found inside the ribcage (Fig. 2 and Supplementary Fig. S1).

The overall torso is dorsoventrally shallow and fusiform (Fig. 2). Twelve articulated dorsal vertebrae are present. However, we can assume that the two most anterior dorsal vertebrae are missing because fourteen dorsal ribs are preserved on each side. The dorsal centra are spool-shaped and longer than high or wide. Ossified tendons are present along the neural spines. The centra of the posterior dorsal vertebrae are a bit shorter in length, and the lateral surfaces are less concave than the anterior ones. No sacral vertebrae are preserved posterior to the dorsal vertebrae. Nine fused posterior dorsal vertebrae from the sixth to 14th are also fused with their ribs to form a long presacral rod. The dorsal ribs increase progressively in size posteriorly from the first to the 11th. These ribs each have a horizontally expanded, flat dorsal surface with a ventrally deep head, which gives the rib a T- or L-shaped proximal cross-section. These ribs also have irregular plate-like bony processes that extend posteriorly from the mid-shaft regions. The processes overlap the lateral surfaces of up to three following ribs. In the third to sixth dorsal ribs, these processes are co-ossified to each other, forming a large lateral plate. The 12th to 14th dorsal ribs are rod-like and smaller and anteroposteriorly narrower than the 11th dorsal ribs. A parasacral rib (*sensu*^16^) is present posterior to the 14th dorsal ribs. These are also slender, similar to the 12th to 14th dorsal ribs, and the lateral portion is posteriorly arched where it contacted the ilium posterolaterally. The most anterior sacral rib is preserved on the right side only. This robust rib is horizontal and fused laterally to the ilium.

Only the left scapula and coracoid are preserved, and a suture line is observable between the two bones (Fig. 2). Although the preserved scapula is fragmentary, the coracoid is moderately complete. A hook-like sternal process is present on the ventral margin of the coracoid. The coracoid foramen is located near the glenoid fossa, which faces posterolaterally and is separated by an open suture from the scapula. The unfused sternal plates are large and triangular. The hourglass-shaped humeri are short and robust, and in each the maximum proximal width is about 50% that of the total length. The deltopectoral crest extends distally to about the middle of the humerus and has a lateral process on the proximal edge, which can be observed on the left side. The crest extends more laterally than anteriorly. The humeral shaft is short and thick. Both rod-like radii are present. The expanded proximal and distal condyles of the radius are similar in size. Both robust ulnae are also preserved and each has a blunt olecranon. The medial process of the ulna is prominent. Five metacarpals are preserved in a shallow arc on both sides. All metacarpals are robust, with expanded proximal articular surfaces and distal condyles. Metacarpals I to IV are similar in size, but metacarpal V is smaller. Five proximal phalanges (I-1 to V-1) are present on the right manus, but only the first proximal phalanx (I-1) is preserved of the left manus. The phalanges are wider distally than proximally.

An undistorted right ilium is preserved, which diverges at an angle of around 28° from the midline of the body (Fig. 2). It is long with a blade-like preacetabular process extending anterolaterally. The closed acetabulum is level with the medially positioned first sacral rib. A short buttress-like postacetabular process extends posterolaterally from the acetabulum. The femur is uncrushed but only preserved with posteriorly expanded distal condyles, of which the lateral condyle projects more ventrally than the medial one. The stout tibiae are straight, and each has an anteroposteriorly expanded proximal condyle. Both of the long and narrow fibulae are preserved, although the distal portion of the right one is missing. They are straight or slightly curved. Left metatarsals II to IV, and right metatarsals II and III are preserved. They are elongate and somewhat hour-glass shaped. Metatarsal III is only slightly longer than metatarsals II and IV. Pedal phalanges II-1 to -3, III-1, and IV-1 to -3 are preserved on the left side, whereas III-1 to -3 and IV-1 to -3 are present on the right side. The phalangeal count is 0-3-3-3-0. Based on the well-preserved unguals on the left pes, the unguals are longer than wide. The distal portion of the unguals on the right pes are poorly preserved.

Large, dorsoposteriorly bent spine-like Type 1 osteoderms (*sensu*^17^) are present in a row along the lateral side of the body (Fig. 2). The fifth to 12th lateral osteoderms are preserved on the right side, whereas the first to eighth are preserved on the left side. The most anterior Type 1 osteoderm is positioned above the forearm. The osteoderms increase in size from the first to seventh. The eighth Type 1 osteoderm is about half the volume of the seventh one. Posteriorly from here, the osteoderms increase again in size from the eighth to the 12th. The most posterior osteoderm is lateral to the preacetabular process of the ilium. A slightly compressed, keeled Type 7 osteoderm with a backswept tip was collected in the central region of the right side of the trunk, slightly dorsal to the lateral Type 1 osteoderms (Fig. 3b,c). Its dorsal surface is rugose. A total of six small, nodular Type 6 osteoderms are present on the ventral portion of the trunk (Fig. 2). Five of them are observable on the left side, whereas three can be seen on the right side. On the left side, one of these osteoderms is between the first and second dorsal ribs, whereas the other four are situated slightly ventromedially along with the large lateral Type 1 osteoderms. On the left side, one is situated between the fifth and sixth dorsal ribs, whereas the other two are positioned near the distal tip of the ninth dorsal rib and behind the 11th dorsal rib, respectively. Another single large flat Type 7 osteoderm (longitudinal length: about 125 mm, transverse width: about 100 mm) with a slightly concave ventral surface is present on the right side of the ribcage behind the seventh rib (Fig. 2). Only the ventral side of this osteoderm is observable at present. Three small, conical, and circular based Type 4 osteoderms with small neurovascular canals on the external surface are also preserved: two within the ribcage and one beneath the pelvic region (Figs. 2, 3d-k). Medium-sized, trapezoidal or oval, keeled Type 5 osteoderms cover the lateral side of the forelimb: thirteen on the right and twelve on the left side (Fig. 2).

## Discussion

**Figure 4:**
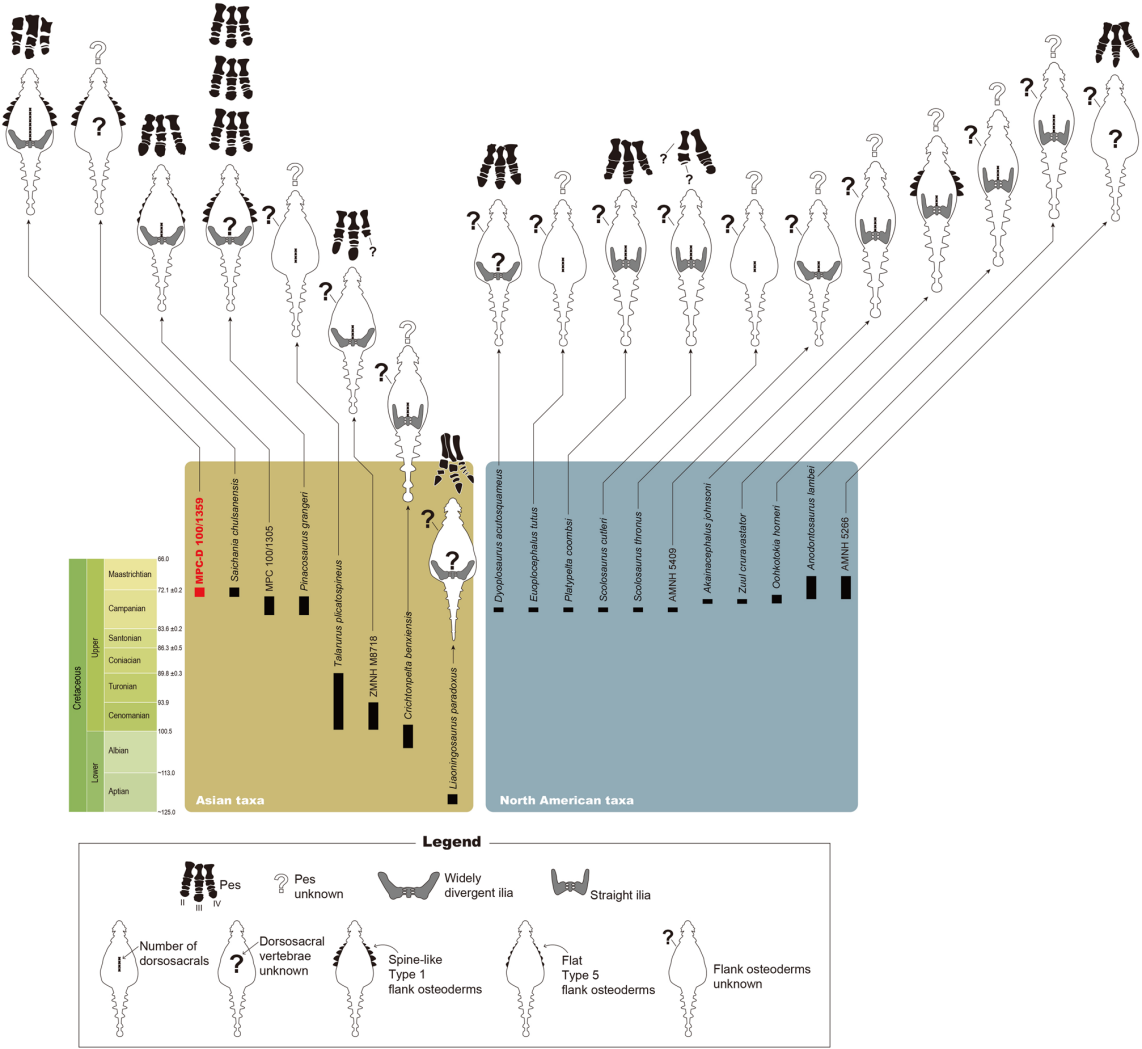
The temporal range of all known ankylosaurids with either well-preserved sacral rods and pelvic girdles, pedes, or flank osteoderms. Adobe Illustrator CC (version 24.0.1, https://www.adobe.com/kr/products/illustrator.html) was employed to produce figure.

MPC-D 100/1359 has nine sacrodorsal vertebrae (Fig. 2), the highest number known for ankylosaurs. Three to four sacrodorsals is the typical number for ankylosaurids, except *Anodontosaurus lambei*, which has five sacrodorsals^2,16,18–22^ (Fig. 4). Furthermore, the presence of broad but irregular, plate-like, bony intercostal processes are rarely seen on the mid-shafts of the dorsal ribs. This feature is shared only with *Saichania* from the Baruungoyot Formation^3^. The extensively fused vertebral series with unique dorsal ribs must have reduced the flexibility of the trunk. This character has been interpreted as possibly important in the evolution of bracing against tail movements^23,24^. However, the reason that Asian ankylosaurids had less flexible trunks compared to North American taxa is unclear. Although no ankylosaurid tail has been described from the Baruungoyot Formation in detail, an almost complete tail (ZPAL MgD I/113) from the Nemegt Formation (upper Campanian-lower Maastrichtian) suggests that some Asian ankylosaurids had more elongate tails than those of North American taxa^17^. The high rigidity of the trunk in some Asian ankylosaurids might have functioned as a buttress for the longer tail.

The pedal phalangeal formula of MPC-D 100/1359 (0–3-3–3-0) is the same to those of one *Pinacosaurus grangeri* specimen (MPC 100/1320) and MPC 100/1305 (Ankylosauridae indet.) from the Djadokhta Formation (Campanian), but differs from that of earlier Asian ankylosaurids such as *Liaoningosaurus* (0-3-4-5-0) and the majority of *P. grangeri* specimens (0-3-4-4-0 and 0-3-3-4-0) as well as North American ankylosaurid taxa (0-3-4-3/4–0)^4,10,22,25–28^ (Fig. 4). Although pedal phalangeal formulae can differ between individuals within same species, as in *P. grangeri*^10^, the phalangeal formula shows that the pedal phalanges, especially the second and third digits, of Asian ankylosaurids decreased in number through time. Decrease in the number of phalanges is recognized in the manus of sauropods as a way to support their greater body masses^29^. It is possible that this transition occurred in the pedes of Asian ankylosaurs in correlation with the ventrally projected lateral condyles of the femora, which are considered as adaptations for weight-bearing^4^ and have been observed in several Asian specimens (e.g., *Chuanqilong*, *Crichtonpelta*, *Talarurus*, MPC 100/1305, and ZMNH M8718)^2,4,18,30,31^. Furthermore, the reduced number of pedal phalanges resulted in less mobility of the limbs^29^. Like the highly rigid trunk, this feature might have also functioned as a less-flexible buttress for a long tail.

MPC-D 100/1359 is the fourth ankylosaurid specimen to be described with in situ flank osteoderms. The previously described specimens are MPC 100/1305 and PIN 614 (*P. grangeri*) from the Djadokhta Formation, and MPC 100/151 (holotype of *Saichania*) from the Baruungoyot Formation^2–4^. Both MPC 100/151 and PIN 614 have spine-like Type 1 osteoderms on the flanks as in MPC-D 100/1359. Instead of Type 1 osteoderms, however, keeled rhomboid Type 5 osteoderms are present on the sides of MPC 100/1305^4^ (Fig. 4), suggesting that there were at least two forms of flank armor within Asian ankylosaurids.

Few bones of MPC-D 100/1359 are distorted, and none are dislocated from the death posture, which suggests there was no post-mortem transportation. Feeding traces of dermestid beetles are present on the bone surfaces (Fig. 3l,m). Because rapid and deep burial prevents the activity of dermestid beetles, Fanti et al.^32^ suggested that the presence of these traces might be evidence of exposure of the body, allowing the development of a dermestid colony within the carcass. Some dorsal osteoderms (Type 4 and 7) are preserved inside the ribcage and the pelvic region (Figs. 2, 3d-k), which seem to have sunk into the body during decomposition before the final burial. On the other hand, the spine-like osteoderms on the flanks and pelvic area are preserved in place with feeding traces of dermestid beetles present on the ventral surfaces. This preservation difference implies that the lower part of the body of MPC-D 100/1359, slightly beneath the lateral osteoderms, was exposed at some point and was scavenged by beetles, before the sediments finally covered the rest of the body. The five isolated theropod phalanges collected inside the trunk must have entered the ribcage from above before the final burial of this specimen (Fig. 2 and Supplementary Fig. S1).

Several ankylosaur specimens preserved in a similar “resting posture” to MPC-D 100/1359 were previously reported from the Djadokhta (Campanian) and Baruungoyot (middle-upper Campanian) formations of Mongolia^2,3,10,33,34^, and the Bayan Mandahu Formation (Campanian) of China^10,35^. Because all of these specimens were discovered in rock units that were deposited in semiarid to arid climates in eolian environments, it was interpreted that they were buried alive in dust storms or rainstorm episodes^36,37^. MPC-D 100/1359 also perished in situ, possibly due to famine, dust storms, or any other possible number of reasons.

A few authors have previously remarked that ankylosaurids show adaptations for digging. Maleev^2^ first hypothesized that “*Syrmosaurus viminicaudus*” (= *P. grangeri*) dug with anteroposterior body movements and covered up the lateral sides of their bodies with sediments using both fore- and hind limbs to leave the dorsal parts of their bodies exposed, similar to modern horned lizards (*Phrynosoma*). Maryañska^3^ supported this hypothesis based on the dorsoventrally flattened body shape and keeled dermal armor on lateral sides of the body of *Saichania*. Furthermore, Coombs^38^ concluded by reconstructing their forelimb muscles that digging habits were possible for ankylosaurids. No one has discussed the possibility of whether ankylosaurids were adapted for digging ever since.

Most ankylosaurids, including MPC-D 100/1359, show characteristics of digging, such as large acromion processes on the scapulae, robust humeri with well-developed deltopectoral crests, radii shorter than humeri, short and robust ulnae with well-developed olecranon processes, manus shorter than radii (terminal phalanges excluded), short and wide metacarpals and proximal phalanges, low intermembral indices (< 70), and short or absent pubic symphyses^39–43^. Although the forelimb characters are also generally assumed to be related to weight-bearing^38^, low intermembral indices and the reduction or absence of pubic symphyses are considered two of the most indicative features of digging^44^. Ankylosaurids also have a dorsoventrally flattened and fusiform bodies with anteroposteriorly broadened dorsal ribs. This is an adaptation linked to counteracting the transverse bending force caused by the digging movements of the forelimbs^43,45^. Additionally, the extensive fusion of vertebrae and dorsal ribs, and the decreased numbers of pedal phalanges observed in MPC-D 100/1359 appear to be related to digging as well. The high rigidity of the trunk may have stabilized the body during digging with forelimbs. The reduction of mobility in the hind limbs – caused by the decreased number of pedal phalanges – may have been suitable for anchoring ankylosaurs when they were digging^40^.

Coombs^38^ concluded that the ankylosaurid manus does not show special modifications for digging behaviours, because the hoof-like manual unguals of ankylosaurids are different from the long, deep, and claw-like unguals of most digging animals. However, bony ungual length or curvature may not correlate well with the ecology of animals^46^. The metacarpals of ankylosaurid forefeet, including those of MPC-D 100/1359, are arranged in a shallow arc (Fig. 2) that can increase the stiffness of the forefeet^3,10,45^. This shape gives the manus a shovel or trowel-like appearance. Even though ankylosaurids did not have the claw-like unguals, therefore, it seems that they may have been able to dig soft substrate with the stiff trowel-like manus.

Tumanova^47^ rejected the possibility of digging in ankylosaurids because of their large body sizes. However, body size cannot be used to disqualify digging abilities in ankylosaurs because similar-sized, large animals such as mylodontid ground sloths were capable of digging^48^. Although mylodontids were capable of dwelling in underground spaces^49^, it is doubtful that ankylosaurids could have done the same. To date, no paleoburrows attributable to ankylosaurs have been reported from ankylosaurid-bearing strata. Moreover, the stiffened elongated tail of ankylosaurids would not be suitable for dwelling in underground spaces.

Anatomical features discussed in this paper could be good supporting evidence for the surface digging ability of ankylosaurids. Taking advantage of digging, ankylosaurids may have been able to dig out roots for food, dig wells to reach subsurface water, or dig into the sediments so they could find supplementary minerals to consume as modern African elephants (*Loxodonta*) do today^50^.

It was long assumed that ankylosaurids might have hunkered down to defend their relatively soft undersides from predators^51^. Crouching down in the shallow pits they made might have helped them protect their limbs and vulnerable belly parts, and to anchor their bodies so as not to get turned over by large predators. Extant horned lizards (*Phrynosoma*) have a similar flat body and lateral fringe scales to those of ankylosaurids, which makes it difficult for predators to detect their silhouettes when partially buried or at rest on flat substrates^52–54^. Ankylosaurids may have utilized a similar strategy.

## Methods

The specimen (MPC-D 100/1359) was divided into three separate plaster jackets for shipment to a laboratory in Hwaseong City of South Korea for preparation in 2012. In the lab, all jackets were opened from the bottom to expose the ventral side of the skeleton. After preparation, it was returned to Mongolia in 2016, where it is permanently held in the Institute of Paleontology in Ulaanbaatar, Mongolia.

All measurements were taken using a measuring tape and a digital caliper. Comparisons to other ankylosaurid taxa were made by examining some specimens in the Institute of Paleontology, Mongolian Academy of Sciences, Mongolia, or were extracted from published literature. Phalangeal formulae were based on Padian^54^. Osteoderm types correspond to the terms used by Arbour et al.^18^.

## Supplementary Information

Supplementary Information
