# Supplementary material for: A new ankylosaurid skeleton from the Upper Cretaceous Baruungoyot Formation of Mongolia: its implications for ankylosaurid postcranial evolution
Source: Sci Rep. 2021 Mar 18;11:4101. doi: 10.1038/s41598-021-83568-4 (PMC7973727; doi:10.1038/s41598-021-83568-4)
Supplement: Supplementary file 1 — Supplementary Information [file 41598_2021_83568_MOESM1_ESM.docx]

**Supplementary information**

**A new ankylosaurid skeleton from the Upper Cretaceous Baruungoyot Formation of Mongolia: its implications for ankylosaurid postcranial evolution**

Jin-Young Park^1^, Yuong-Nam Lee^1,*^, Philip J. Currie^2^, Michael J. Ryan^3,4^, Phil Bell^5^, Robin Sissons^2^, Eva B. Koppelhus^2^, Rinchen Barsbold^6^, Sungjin Lee^1^ and Su-Hwan Kim^1^

^1^ School of Earth and Environmental Sciences, Seoul National University, Seoul, 08826, South Korea

^2^ Department of Biological Sciences, CW 405 Biological Sciences Building, University of Alberta, Edmonton, AB T6G 2E9, Canada

^3^ Department of Earth Sciences, Carleton University, 2125 Herzberg Building, 1125 Colonel By Drive, Ottawa, Ontario, K1S 5B6, Canada

^4^ Department of Palaeobiology, Canadian Museum of Nature, P.O. Box 3443, Station ‘D’, Ottawa, Ontario, K1P 6P4, Canada

^5^ Palaeoscience Research Centre, University of New England, Armidale 2351, New South Wales, Australia

^6^ Institute of Paleontology, Mongolian Academy of Sciences, Box-46/650, Ulaanbaatar 15160, Mongolia

**Contents**

1) Comparisons of MPC-D 100/1359 to other Asian ankylosaurids

2) Supplementary figure

3) Supplementary tables

4) Supplementary references

**1. Comparisons of MPC-D 100/1359 to other Asian ankylosaurids**

**Institutional abbreviations**

**MPC**, Mongolian Paleontological and Geological Institute, Mongolian Academy of Sciences, Ulaanbaatar, Mongolia; **ZMNH**, Zhejiang Museum of Natural History, Hangzhou, Zhejiang, People's Republic of China.

***Bissektipelta archibaldi*:** No overlapping portions are present between this taxon and MPC-D 100/1359.

***Chuanqilong chaoyangensis*:** This taxon is similar to MPC-D 100/1359 by having widely divergent ilia, a ventrally projected lateral condyle on the femur, and triangular unguals on the pes. However, it differs in having a gracile ulna [1].

***Crichtonpelta benxiensis*:** This taxon is similar to MPC-D 100/1359 by having fifteen dorsal vertebrae, widely divergent ilia, and a ventrally projected lateral condyle on the femur. However, it differs by having eleven free dorsal vertebrae and four dorsosacral vertebrae [2].

***Gobisaurus domoculus*:** No comparable postcranial materials are described in scientific literature.

***Jinyunpelta sinensis*:** There are no noticeable differences between this taxon and MPC-D 100/1359, although only limited postcranial materials of the former were described in the scientific literature.

***Liaoningosaurus paradoxus*:** This taxon is similar to MPC-D 100/1359 by having widely divergent ilia, triangular pedal unguals. However, it differs by having both lateral and medial condyles on the femur in the same horizon and a pedal phalangeal count 0-3-4-5-0 [3].

***Minotaurasaurus ramachandrani*:** No postcranial materials are described in scientific literature.

***Pinacosaurus grangeri*:** This taxon is similar to MPC-D 100/1359 by having seven free dorsal vertebrae, widely divergent ilia, and Type 1 osteoderms present along the flanks and the preacetabular process of the ilium. However, it differs by having a preserved pair of fused sternal plates with long narrow lateral processes on the posterolateral edge, a gracile ulna, both lateral and medial condyles on the femur extend to the same level, some specimens having a pedal phalangeal count 0-3-3/4-3/4-0, a hoof-like ungual phalanx Ⅲ-4 on the pes, and up to eighteen Type 1 osteoderms on each side of the flank and up to seven on each side of the pelvic region [4] [5] [6] [7].

***Pinacosaurus mephistocephalus*:** This taxon is similar to MPC-D 100/1359 by having widely divergent ilia [8]. There are no noticeable differences between this taxon and MPC-D 100/1359, although only limited postcranial materials of the former were described in the scientific literature.

***Saichania chulsanensis*:** Comparison with this taxon is mentioned in the main text.

***Shamosaurus scutatus*:** There are no noticeable differences between this taxon and MPC-D 100/1359, because of limited postcranial materials that are described in the scientific literature.

***Talarurus plicatospineus*:** This taxon is similar to MPC-D 100/1359 in having a robust ulna, and a lateral condyle on the femur that more projects ventrally than the medial one. However, it differs by having four dorsosacral vertebrae [10] [11] [12].

***Tarchia kielanae*:** See main text.

***Tarchia teresae*:** No postcranial materials are described in the scientific literature.

***Tsagantegia longicranialis*:** No postcranial materials are known from this taxon.

***Zaraapelta nomadis*:** See main text.

**MPC 100/1305:** This specimen is a nearly complete skeleton without a skull from the Djadokhta Formation (Campanian). Only two ankylosaurine taxa, *Minotaurasaurus ramachandrani* and *Pinacosaurus grangeri*, are known from the Djadokhta Formation [4] [5] [6] [7] [13] [14] [15] [16], and Arbour and Currie [17] considered this specimen as Ankylosauridae indet. or cf. *Pinacosaurus*. As mentioned previously, no postcranial elements of *Minotaurasaurus* have been described, so comparing MPC 100/1305 to this taxon is currently not possible. On the other hand, MPC 100/1305 differs from *P. grangeri* by having seven free dorsal vertebrae, a fused pair of sternal plates with an anterior notch and a smooth lateral margin, a robust ulna, and a more distally projected lateral condyle on the femur [6] [7] [17] [18]. Therefore, it is certain that MPC 100/1305 is not *P. grangeri*, but possibly *Minotaurasaurus*. However, new *Minotaurasaurus* specimens with both cranial and postcranial portions are needed further to clarify the taxonomic identification of MPC 100/1305.

MPC 100/1305 is similar to MPC-D 100/1359 by having a robust ulna, a ventrally projected lateral condyle on the femur, a pedal phalangeal count 0-3-3-3-0, and large Type 1 osteoderms laterally placed to the preacetabular process of the ilium. However, it differs by having ten free dorsal vertebrae, four dorsosacral vertebrae, fused pair of sternal plates with an anterior notch and a smooth lateral margin, and Type 5 osteoderms laterally placed between the forearm and the ilia [18].

**ZMNH M8718:** This specimen was excavated from the Chaochuan Formation (Albian–Cenomanian) and formally known as “*Zhejiangosaurus lishuiensis*” [19] [20]. ZMNH M8718 includes dorsosacral and sacral vertebrae, fourteen caudal vertebrae, both ilia, a single pubis, complete hindlimbs and pes, and some unidentified bone fragments [19]. It is similar to MPC-D 100/1359 by having widely divergent ilia, a slightly ventrally projected lateral condyle on the femur. However, it differs by having six dorsosacral vertebrae, a pedal phalangeal count 0-3-4-5-0, and a hoof-like ungual phalanx Ⅲ on the pes [19] [20].

**2. Supplementary figure**


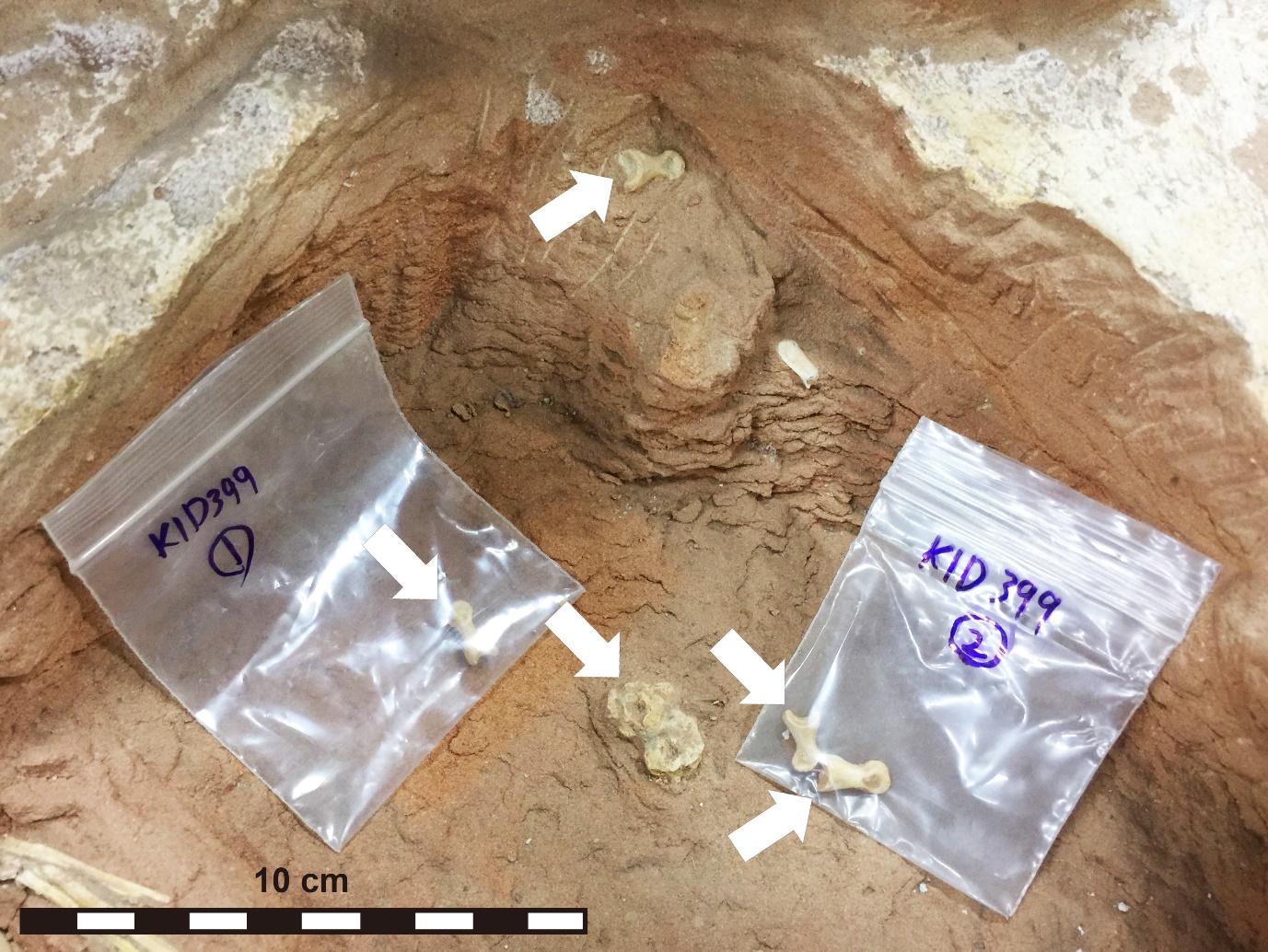


**Supplementary figure S1.** Five isolated theropod phalanges (marked by white arrows) that were collected inside the ribcage of the new ankylosaurid postcranial specimen (MPC-D 100/1359).

**3. Supplementary tables**

**Supplementary table 1.** Measurements (mm) of vertebrae of MPC-D 100/1359. Asterisk refers incomplete measurement due to damage.

| Element | Length of centrum | Width of anterior surface of centrum | Width of posterior surface of centrum |
| --- | --- | --- | --- |
| 4^th^ dorsal | 116 | 4* | 34* |
| 5^th^ dorsal | 120 | 37* | 24* |
| 6^th^ dorsal | 111 | 51* | 49* |
| 7^th^ dorsal | 128 | 91 | 61* |
| 8^th^ dorsal | 130 | 37* | 79 |
| 9^th^ dorsal | 103 | 45* | 53* |
| 10^th^ dorsal | - | 75 | 8 |
| 11^th^ dorsal | 118* | 79 | - |
| 12^th^ dorsal | 122* | 72 | 34* |
| 13^th^ dorsal | 128 | 68* | 77 |
| 14^th^ dorsal | 115 | 77 | 77 |
| 15^th^ dorsal | 122 | 86 | 81 |
| 1^st^ sacral | 98* | 76 | - |

**Supplementary table 2.** Measurements (mm) of pectoral girdle and forelimbs of MPC-D 100/1359. Asterisks refer incomplete measurement due to damage.

| Element |  | Measurements |
| --- | --- | --- |
| Left scapula | Length | - |
|  | Proximal height | 91* |
|  | Medial height | - |
|  | Distal height | - |
| Left coracoid | Length | 218 |
|  | Proximal height | 129* |
|  | Medial height | 105* |
|  | Distal height | 91* |
| Left humerus | Length | 322* |
|  | Proximal width | 169 |
|  | Medial width | 46* |
|  | Distal width | - |
| Right humerus | Length | 372 |
|  | Proximal width | 186 |
|  | Medial width | - |
|  | Distal width | 50* |
| Left ulna | Length | 123* |
|  | Proximal width | - |
|  | Medial width | 67* |
|  | Distal width | 94 |
| Right ulna | Length | 300 |
|  | Proximal width | 158* |
|  | Medial width | 81 |
|  | Distal width | 97 |
| Left radius | Length | 233 |
|  | Proximal width | 91 |
|  | Medial width | 50 |
|  | Distal width | 91 |
| Right radius | Length | 241 |
|  | Proximal width | 50* |
|  | Medial width | 31* |
|  | Distal width | 88 |
| Left sternum | Anteroposterior length | 228* |
|  | Mediolateral width | 62* |
| Right sternum | Anteroposterior length | 273 |
|  | Mediolateral width | 225* |

**Supplementary table 3.** Measurements (mm) of both manus of MPC-D 100/1359. Asterisks refer incomplete measurement due to damage.

| Element | Length | Proximal width | Distal width |
| --- | --- | --- | --- |
| Left metacarpal Ⅰ | 75 | 50* | 57 |
| Right metacarpal Ⅰ | 70 | 43 | 46 |
| Left metacarpal Ⅱ | 70 | 56 | 63 |
| Right metacarpal Ⅱ | 72 | 54 | 55 |
| Left metacarpal Ⅲ | 75 | 58 | 30* |
| Right metacarpal Ⅲ | 79 | 53 | 46 |
| Left metacarpal Ⅳ | 73 | 42 | 33* |
| Right metacarpal Ⅳ | 80 | 45 | 46 |
| Left metacarpal Ⅴ | 25* | - | - |
| Right metacarpal Ⅴ | 65 | 38 | 28 |
| Left manus phalanx Ⅰ-1 | 36 | 26* | 16* |
| Right manus phalanx Ⅰ-1 | 31 | 33 | 36 |
| Right manus phalanx Ⅱ-1 | 26 | 34 | 40 |
| Right manus phalanx Ⅲ-1 | 27 | 37 | 46 |
| Right manus phalanx Ⅳ-1 | 31 | 30 | 34 |
| Right manus phalanx Ⅴ-1 | 12 | 16 | 19 |

**Supplementary table 4.** Measurements (mm) of pelvic girdle and hindlimbs of MPC-D 100/1359. Asterisks refer incomplete measurement due to damage.

| Element |  | Measurements |
| --- | --- | --- |
| Right illium | Length | 769 |
|  | Length of preacetabular process | 558 |
|  | Length of postacetabular process | 55* |
| Left femur | Length | 174* |
|  | Proximal width | - |
|  | Medial width | - |
|  | Distal width | 183 |
| Right femur | Length | 365* |
|  | Proximal width | - |
|  | Medial width | 87* |
|  | Distal width | 179 |
| Left tibia | Length | 293* |
|  | Proximal width | - |
|  | Medial width | 58* |
|  | Distal width | 101 |
| Right tibia | Length | 230 |
|  | Proximal width | - |
|  | Medial width | 62* |
|  | Distal width | 120 |
| Left fibula | Length | 210* |
|  | Proximal width | 74 |
|  | Medial width | 39 |
|  | Distal width | - |
| Right fibula | Length | 282 |
|  | Proximal width | 71 |
|  | Medial width | 41 |
|  | Distal width | 50 |

**Supplementary table 5.** Measurements (mm) of both pedes of MPC-D 100/1359. Asterisks refer incomplete measurement due to damage.

| Element | Length | Proximal width | Distal width |
| --- | --- | --- | --- |
| Left metatarsal Ⅱ | 82 | - | 67 |
| Right metatarsal Ⅱ | 73* | - | 59 |
| Left metatarsal Ⅲ | 126 | - | 60* |
| Right metatarsal Ⅲ | 127 | 65* | 71 |
| Right metatarsal Ⅳ | 69 | 67 | 88 |
| Left pes phalanx Ⅱ-1 | 47 | 60* | 65 |
| Right pes phalanx Ⅱ-1 | 58 | 72 | 55* |
| Left pes phalanx Ⅱ-2 | 12 | 32 | 31 |
| Right pes phalanx Ⅱ-2 | 12 | 40 | 36 |
| Left pes phalanx Ⅱ-3 | 65 | 32* | 34 |
| Right pes phalanx Ⅱ-3 | 37* | 43 | - |
| Left pes phalanx Ⅲ-1 | 50 | 62 | 57 |
| Right pes phalanx Ⅲ-1 | 67 | 71 | - |
| Left pes phalanx Ⅲ-2 | 12 | 46 | 36 |
| Left pes phalanx Ⅲ-3 | 65 | 43 | 34 |
| Right pes phalanx Ⅳ-1 | 39 | 68 | 70 |
| Right pes phalanx Ⅳ-2 | 20 | 51 | 56 |
| Right pes phalanx Ⅳ-3 | 45 | 54 | 29 |

**4. Supplementary references**

[1] Han, F., Zheng, W., Hu, D., Xu, X. & Barrett, P. M. A new basal ankylosaurid (Dinosauria: Ornithischia) from the Lower Cretaceous Jiufotang Formation of Liaoning Province, China. *PLoS ONE* **9**, e104551; 10.1371/journal.pone.0104551 (2014).

[2] Lü, J., Ji, Q., Gao, Y. & Li, Z. A new species of the ankylosaurid dinosaur *Crichtonsaurus* (Ankylosauridae : Ankylosauria) from the Cretaceous of Liaoning province, China. *Acta. Geol. Sin.* **81**, 883–897 (2007).

[3] Xu, X., Wang, X.-L. & You, H.-L. A juvenile ankylosaur from China. *Sci. Nat.* **88**, 297–300 (2001).

[4] Maleev, E. A. The armored dinosaurs of the Cretaceous Period in Mongolia (Family Syrmosauridae). *Trudy Paleontologicheskogo Instituta Akademiy Nauk SSSR* **48**, 142–170 (1954).

[5] Maryañska, T. New data on the skull of *Pinacosaurus grangeri* (Ankylosauria). *Palaeontol. Pol.* **25**, 45–53 (1971).

[6] Currie, P. J., Badamgarav, D., Koppelhus, E. B., Sissons, R. & Vickaryous, M. K. Hands, feet, and behaviour in *Pinacosaurus* (Dinosauria: Ankylosauridae). *Acta Palaeontol. Pol.* **56**, 489–504 (2011).

[7] Burns, M. E., Tumanova, T. A. & Currie, P. J. Postcrania of juvenile *Pinacosaurus grangeri* (Ornithischia: Ankylosauria) from the Upper Cretaceous Alagteeg Formation, Alag Teeg, Mongolia: implications for ontogenetic allometry in ankylosaurs. *J. Paleontol.* **89**, 168–182 (2015).

[8] Godefroit, P., Pereda-Suberbiola, X., Li, H. & Dong, Z.-M. A new species of the ankylosaurid dinosaur *Pinacosaurus* from the Late Cretaceous of Inner Mongolia (P. R. China). *Bull. Inst. Roy. Sci. Nat. Belgique* **69** (Suppl. B), 17–36 (1999).

[9] Tumanova, T. A. Armoured dinosaurs from the Cretaceous of Mongolia in *The Age of Dinosaurs in Russia and Mongolia* (eds. Benton, M. J., Shishkin, M. A., Unwin, D. M. & Kurochkin, E. N.) 517–532 (Cambridge University Press, 2000).

[10] Maleev, E. A. New ankylosaur of the Upper Cretaceous of Mongolia. *Doklady Akademii Nauk SSSR* **87**, 273–276 (1952).

[11] Maleev, E. A. The armored dinosaurs of the Cretaceous Period in Mongolia (Family Syrmosauridae). *Doklady Akademii Nauk SSSR* **48**, 142–170 (1956).

[12] Maryañska, T. Ankylosauridae (Dinosauria) from Mongolia. *Palaeontol. Pol.* **37**, 85–151 (1977).

[13] Gilmore, C. W. On the dinosaurian fauna of the Iren Dabasu Formation. *Bull. Am. Mus. Nat. Hist.* 67, 23–78 (1933).

[14] Hill, R. V., Witmer, L. M. & Norell, M. A. A new specimen of *Pinacosaurus grangeri* (Dinosaur: Ornithischia) from the Late Cretaceous of Mongolia: ontogeny and phylogeny of ankylosaurs. *Am. Mus. Novit.* **3395**, 1–29 (2003).

[15] Dingus, L. *et al*. The geology of Ukhaa Tolgod (Djadokhta Formation, Upper Cretaceous, Nemegt Basin, Mongolia). *Am. Mus. Novit.* **3616**, 1–40 (2008).

[16] Miles, C. A. & Miles, C. J. Skull of *Minotaurasaurus ramachandrani*, a new Cretaceous ankylosaur from the Gobi Desert. *Curr. Sci.* **96**, 65–70 (2009).

[17] Arbour, V. M. & Currie, P. J. The taxonomic identity of a nearly complete ankylosaurid dinosaur skeleton from the Gobi Desert of Mongolia. *Cretac. Res.* **46**, 24–30 (2013).

[18] Carpenter, K. *et al*. *Saichania chulsanensis* (Ornithischia, Ankylosauridae) from the Upper Cretaceous of Mongolia. *Palaeontographica, Abt. A* **294**, 1–61 (2011).

[19] Lü, J. *et al*. New nodosaurid dinosaur from the Late Cretaceous of Lishui, Zhejiang Province, China. *Acta. Geol. Sin.* **81**, 344–350 (2007).

[31] Arbour, V. M. & Currie, P. J. Systematics, phylogeny and palaeobiogeography of the ankylosaurid dinosaurs. *J. Syst. Palaeontol.* **14**, 385–444 (2016).
